# Predicting outcomes of expectant and medical management in early pregnancy miscarriage using machine learning to develop and validate multivariable clinical prediction models

**DOI:** 10.1186/s12884-025-07283-y

**Published:** 2025-02-28

**Authors:** Sughashini Murugesu, Kristofer Linton-Reid, Emily Braun, Jennifer Barcroft, Nina Cooper, Margaret Pikovsky, Alex Novak, Nina Parker, Catriona Stalder, Maya Al-Memar, Srdjan Saso, Eric O. Aboagye, Tom Bourne

**Affiliations:** 1 Queen Charlotte’s and Chelsea Hospital, Imperial College, London, W12 0HS UK; 2 Department of Metabolism, Digestion and Reproduction, Imperial College London, Du Cane Road, London, W12 0NN UK; 3 Department of Cancer and Surgery, Imperial College London, London, UK; 4 Department of Obstetrics and Gynaecology, University Hospitals Leuven, Leuven, Belgium

**Keywords:** Miscarriage, Expectant management, Medical management, Machine learning

## Abstract

**Objective:**

To determine whether readily available patient, ultrasound and treatment outcome data can be used to develop, validate and externally test two machine learning (ML) models for predicting the success of expectant and medical management of miscarriage respectively.

**Methods:**

A retrospective, multi-site study of patients opting for expectant or medical management of miscarriage was undertaken. A total of 1075 patients across two hospital early pregnancy units were eligible for inclusion. Data pre-processing derived 14 features for predictive modelling. A combination of eight linear, Bayesian, neural-net and tree-based machine learning algorithms were applied to ten different feature sets. The area under the receiver operating characteristic curve (AUC) scores were the metrics used to demonstrate the performance of the best performing model and feature selection combination for the training, validation and external data set for expectant and medical management separately.

**Results:**

Parameters were in the majority well matched across training, validation and external test sets. The respective optimum training, validation and external test set AUC scores were as follows in the expectant management cohort: 0.72 (95% CI 0.67,0.77), 0.63 (95% CI 0.53,0.73) and 0.70 (95% CI 0.60,0.79) (Logistic Regression in combination with Least Absolute Shrinkage and Selection Operator (LASSO)). The AUC scores in the medical management cohort were 0.64 (95% CI 0.56,0.72), 0.62 (95% CI 0.45,0.77) and 0.71 (95% CI 0.58,0.83) (Logistic Regression in combination with Recursive Feature Elimination (RFE)).

**Conclusions:**

Performance of our expectant and medical miscarriage management ML models demonstrate consistency across validation and external test sets. These ML methods, validated and externally tested, have the potential to offer personalised prediction outcome of expectant and medical management of miscarriage.

**Supplementary Information:**

The online version contains supplementary material available at 10.1186/s12884-025-07283-y.

## Introduction

Miscarriage is the most common pregnancy complication, affecting 1 in 4 women. Approximately 23 million miscarriages occur every year globally, or 44 per minute [1]. Miscarriage can lead to significant psychological and physical morbidity in women and their partners [2, 3]. Further to the trauma of diagnosis, women with missed or incomplete miscarriages, are then left with the decision regarding how they want to manage their pregnancy loss. The options include expectant, medical or surgical management. However, provision of accurate outcome prediction to aid in the decision making is lacking. A clinical decision support tool which generates personalised outcome predictions for each miscarriage management option, may alleviate some of this burden. A machine learning based model could provide each individual a personalised chance of success with expectant and medical management respectively.

Expectant management follows the natural progression of miscarriage and is an attractive option for women who wish to avoid all forms of medical intervention in the treatment of miscarriage. Expectant management is relatively inexpensive and safe, but quoted failure rate varies between 10 and 75% [4–7]. Randomized trials comparing medical treatment, most often misoprostol, with expectant management or placebo show substantial variation in success rates defined as complete miscarriage without surgical intervention [4, 8–13]. A challenge during the decision to opt for expectant management of miscarriage is the lack of defined features which reliably predict the likelihood of a complete spontaneous resolution of pregnancy.

National Institute of Clinical Excellence, National guideline UK [14] recommends expectant management for 7 to 14 days as the first-line strategy from women with a confirmed diagnosis of miscarriage [14]. However, there are caveats to this: if the women has a risk of haemorrhage or evidence of infection, or if she has previous adverse and/or traumatic experience associated with pregnancy. The guideline goes on to recommend offering medical management if expected management is not acceptable to the woman. Thus, demonstrating the importance of patient choice in determining miscarriage management pathway. There are no references to ultrasound findings or patient demographic features in guiding miscarriage management choice.

One previous study [15] used clinical data to develop a prediction model with multinomial logistic regression (MLR) for expectant management of miscarriage outcome. This model achieved an AUC of 0.796 with three clinical features used to build a binary MLR model to predict the probability of successful expectant management of first trimester miscarriage. Another study [16] has developed a model to predict medical management of miscarriage outcomes. Hamel 2022, developed a multivariable logistic regression model, using four clinical features, with an AUC of 0.67 to distinguish between success and failure of medical management in miscarriage [16].

The aim of this study is to harness the use of multiple machine learning methods applied to a range of readily available patient demographic, symptom and ultrasound features. These features were selected following systematic review and discussion with the clinical team of likely relevant features. The use of multiple machine learning methods and feature selection techniques facilitates the selection of the best performing models for the successful prediction of expectant and medical miscarriage management in the first trimester. Such models may have use as a clinical decision support tool, offering personalised outcome prediction data to guide the best management approach, alongside patient choice to optimise the chance of successful miscarriage management. These models could potentially reduce the risks associated with failed expectant or medical management, whilst avoiding unnecessary surgical intervention in those with a high chance of success.

## Methods

The following patient demographics and clinical parameters were collected: maternal age, parity, previous caesarean section, previous surgical management of miscarriage, bleeding score, pain score, gestational age by last menstrual period (LMP), miscarriage type (missed miscarriage (defined as the absence of ultrasound features or symptoms indicating the process of passing pregnancy tissue has begun) or incomplete miscarriage (defined as non-viable pregnancy where bleeding has begun but pregnancy tissue remains in the uterus)), ultrasound measurement of crown rump length (CRL) (in mm), ultrasound measurement of gestational sac (GS) (mean sac diameter in mm), ultrasound measurement of retained pregnancy tissue (mean diameter in mm), vascularity (colour score 1 (no flow), 2 (minimal flow), 3 (moderate flow) and 4 (very strong flow)), gestational age by CRL (or GS if no CRL) and discrepancy between CRL and LMP. Patients were asked about their symptoms of pain and bleeding using validated symptom scores at each visit [17, 18]. These variables were selected following systematic review of studies evaluating predictors of miscarriage management outcomes and discussion with clinical experts. Successful miscarriage management was defined as follow-up ultrasound findings with an endometrial thickness of less than 15 mm [19]. The data was readily available given this information is collected in routine clinical care of miscarriage patients.

### Ethics statement

HRA REC Reference: 23/PR/0297.

Data was collected retrospectively and annonymised, thus patient consent was not obtained, as per approved study protocol by the Health Research Authority NHS.

### Datasets

We used four independent, novel datasets of patients diagnosed with missed or incomplete miscarriage and counselled regarding management options, resulting in a total of 1075 patients across two hospitals. Each dataset was retrospectively collated from the electronic patient record (EPR) (Cerner) and the ultrasound-reporting database (Astraia).

- Dataset ICL E1 consisted of 565 patients diagnosed with missed or incomplete miscarriage treated at Queen Charlotte’s and Chelsea Hospital, Imperial College Healthcare NHS Trust (QCCH), with expectant management between 01/01/2018 and 30/06/2022.
- Dataset ICL E2 consisted of 158 patients diagnosed with missed or incomplete miscarriage treated at St Mary’s Hospital, Imperial College Healthcare NHS Trust (SMH), with expectant management between 01/01/2021 and 31/12/2021.
- Dataset ICL M1 consisted of 276 patients diagnosed with missed or incomplete miscarriage treated at Queen Charlotte’s and Chelsea Hospital, Imperial College Healthcare NHS Trust (QCCH), with medical management between 01/01/2018 and 30/06/2022 (254 final in model))
- Dataset ICL M2 consisted of 76 patients diagnosed with missed or incomplete miscarriage treated at St Mary’s Hospital, Imperial College Healthcare NHS Trust (SMH), with medical management between 01/01/2021 and 31/12/2021 (69 final in model).

Datasets ICL E1 and ICL M1 were randomly divided into training and validation sets for expectant and medical management of miscarriage respectively. The testing sets were E2 for expectant management and M2 for medical management. These test data sets ring-fenced patients from a separate early pregnancy unit and thus formed a geographically external test set. Consistent with routine clinical management, the primary end point to determine success of treatment was if the management had resulted in complete miscarriage within two weeks from the decision to pursue expectant management or within two weeks of administration of medical management. Patients were excluded if they were lost to follow up or if follow up occurred prior to this two week interval and demonstrated retained pregnancy tissue. This cut-off was agreed by the authors after discussion about how best to standardise the outcome analysis and correlate with current practice and recommendations with regards to follow-up during miscarriage management.

To prepare the data for machine learning, categorical variables were transformed into numeric format using one-hot encoding, which created a binary feature for each category level. Missing clinical data were treated as missing at random and independent of the outcome. These gaps were addressed through imputation with the multiple imputation with chained equations (MICE) package in R [20], using 5 imputed data sets as per the default settings. Convergence checks, including visual inspection of trace plots and evaluation of imputed values across iterations, alongside sensitivity analysis, were conducted to ensure the stability and robustness of the MICE process.

Highly correlated features were identified and removed using the treatment_corr function, applying a threshold of 0.85 as the default indicating a very strong linear or monotonic relationship between features. Retaining both features would introduce redundancy, therefore one feature from each such correlated pair was eliminated (based on Pearson correlation for continuous data and Spearman correlation for categorical data). The threshold of 0.85 based on commonly accepted practices in similar studies, aiming to balance the retention of meaningful predictors with the minimization of multicollinearity [21]. This threshold provides an appropriate balance for this study a lower threshold could potentially reduce collinearity further, however the downside of a lower threshold includes loss of important predictors, particularly those of contextual relevance. Overly aggressive feature elimination may lead to an overly simplistic model that does not capture the complexity of the relationships in the data. This can cause the model to underperform, especially in scenarios where correlated variables jointly contribute to the outcome. Continuous variables were standardized using z-score normalization, to a mean of 0 and a standard deviation of 1, enabling comparability across features and improving model performance by mitigating the influence of differing scales [22]. Z-scores were used to identify outliers, defined as observations exceeding 3 standard deviations from the mean. These outlier values were removed to mitigate the potential influence of extreme values, ensuring that the remaining dataset retained its representativeness and integrity.

### Statistics

Patient demographics and clinical parameters were summarized using medians and ranges for continuous variables, and frequencies with percentages for categorical variables. Comparisons between datasets were conducted using the Wilcoxon rank sum test for categorical data and the Kolmogorov–Smirnov test for continuous data, as presented in Tables 1 and 2.

To prevent bias in dataset assignment for training, validation, and testing, cases from datasets ICL E1 (expectant management) and ICL M1 (medical management) were randomly split into training and validation sets in an 80:20 ratio. Stratification was applied based on the binarized outcome to maintain balanced class distributions. Independent test datasets, ICL E2 (expectant management) and ICL M2 (medical management), were reserved to ensure unbiased external evaluation of the models.

### Feature handling and modelling

Dimensionality reduction is often necessary before modelling to enhance prediction accuracy, mitigate overfitting, and lower computational costs. In this study, we applied eight machine learning algorithms, including linear, Bayesian, neural-net, and tree-based methods, to ten feature sets. These feature sets either underwent no reduction or were processed using one of nine reduction techniques: correlation-based, multivariate linear penalization, or recursive approaches. Hyperparameter optimization was conducted using grid search with 10-fold cross-validation, implemented through the caret package in R, to identify the best-performing model configurations.

F1-scores were calculated for all the model and feature selection combinations. Receiver-Operator Characteristic (ROC) curves and Area Under the Curve (AUC) were generated for the training, validation and external test set results for our best performing model. Ensemble prediction models were then explored by averaging the predictions of the three algorithms with the highest F1-score in the validation set for each particular outcome being predicted. Where the ensemble model was superior, it was selected as the final model for predicting that particular outcome on the external test set.

Caret’s varImp function 10 was used to identify features that contributed most significantly to model performance. It provides a generic method for calculating variable importance by either utilising a model’s native feature importance ranking method or using ROC curve analysis for each feature. It is not possible to do this for averaged predictions across algorithms and so where an ensemble was selected as the final model, this was performed for the component algorithms instead.

### Benchmarking

Benchmarking for this prediction model was not possible given there are no prediction models in use in current clinical practice, for this clinical decision. At present all women experiencing miscarriage in the first trimester with missed miscarriage or incomplete miscarriage are given a non-specific failure rate which varies between 10 and 75% [4–7]. Similarly the literature show wide variation in medical management success rate: between 69% [16] and 80% [23].

## Results

In total 1075 patients were included in the study. 723 miscarriage patients opted for expectant management, and 352 miscarriage patients opted for medical management. Within the expectant management groups 387 had a missed miscarriage and 336 had an incomplete miscarriage diagnosis. Within the medical management groups 332 had a missed miscarriage and 20 had an incomplete miscarriage diagnosis. Of the 723 patients opting for expectant management 346 had a successful outcome and 377 required further management. Within the 352 medical management of miscarriage patients, 150 had a successful outcome and 203 required further management. A more detailed breakdown of feature frequency by cohort is presented in Tables 1 and 2.

**Table 1:** Expectant management cohort descriptive statistics

Parameter	Combined Training & Validation data (*n* = 565)	Test data (*n* = 158)	*P*-value
**Median Maternal Age (**Range)	35 (19–51)	34 (21–46)	0.2041
**Gravida**			0.9946
-1	156	48	
-2	140	41	
-3	98	27	
-4	49	15	
-5	35	13	
-6	19	6	
> 6	23	6	
-NA	45	2	
**Parity**			0.8433
-0	234	72	
-1	186	48	
-2	59	23	
-3	25	5	
-4	10	6	
-5	6	1	
->5	4	2	
-NA	41	1	
**Number of Previous Miscarriage/Termination of Pregnancy**			0.7226
-0	286	88	
-1	129	40	
-2	55	15	
-3	36	8	
->3	13	5	
-NA	46	2	
**Ethnicity**			0.008296
**-**Asian and Asian British	112	35	
-Black, Black British, Caribbean or African	87	26	
-Mixed or multiple ethnic groups	12	7	
-White	195	56	
-Other ethnic group	100	3	
-NA	59	30	
**Previous CS**			0.001879
-Yes	76	28	
-No	392	120	
-NA	97	10	
**Bleeding Score at Presentation**			0.8939
-0	50	6	
-1	44	17	
-2	52	16	
-3	23	7	
-4	44	7	
-NA	352	105	
**Pain Score at Presentation**			0.1273
-0	82	21	
-1	1	0	
-2	7	2	
-3	2	0	
-4	3	2	
-5	7	0	
-6	4	0	
-7	4	0	
-8	2	0	
-9	4	0	
-10	1	1	
-NA	448	133	
**Previous SMM/MVA**			2.125 × 10^− 7^
-yes	33	18	
-No	307	104	
-NA	225	36	
**Diagnosis Type**			0.1806
-Missed	295	92	
-Incomplete	270	66	
**Missed Miscarriage CRL Median (range)(mm)**	9.6 (1.2–56)	14.0 (0.7–54.7)	4.61 × 10^− 5^
**Missed Miscarriage GS Median (range) (mm)**	22.7 (4–62.3)	22.5 (3.2–57)	0.5557
**Gestation of Miscarriage by USS Measurements**			0.5879
-5	6	0	
-6	55	13	
-7	68	12	
-8	62	20	
-9	14	19	
-10	2	7	
-11	3	3	
-12	4	2	
-NA	351	82	
**Gestation of Miscarriage by LMP**			0.4945
< 6	2	0	
-6	19	4	
-7	35	8	
-8	70	23	
-9	97	25	
-10	97	23	
-11	73	24	
-12	61	21	
-13	37	5	
->13	22	9	
-NA	50	16	

**Table 2:** Medical management cohort descriptive statistics

Parameter	Combined Training & Validation data (*n* = 276)	Test data (*n* = 76)	*P*-value
**Median Maternal Age (**Range)	36 (21–48)	35.5 (18–50)	0.2318
**Gravida**			0.02946
-1	70	31	
-2	70	18	
-3	45	14	
-4	26	4	
-5	12	3	
-6	11	2	
> 6	12	2	
NA	30	2	
**Parity**			0.2533
-0	125	40	
-1	71	27	
-2	29	7	
-3	13	1	
-4	7	1	
-5	2	0	
->5	2	0	
-NA	25	0	
**Number of Previous Miscarriage/Termination of Pregnancy**			0.01042
-0	125	52	
-1	68	13	
-2	28	1	
-3	11	4	
->3	12	4	
-NA	32	2	
**Ethnicity**			0.8043
**-**Asian and Asian British	38	19	
-Black, Black British, Caribbean or African	30	7	
-Mixed or multiple ethnic groups	8	5	
-White	121	38	
-Other ethnic group	42	1	
-NA	37	5	
**Previous CS**			0.2566
-Yes	40	59	
-No	179	11	
-NA	57	6	
**Bleeding Score at Presentation**			0.01048
-0	68	7	
-1	17	4	
-2	15	5	
-3	3	2	
-4	3	1	
-NA	170	57	
**Pain Score at Presentation**			0.4932
-0	57	9	
-1	2	0	
-2	2	0	
-3	2	0	
-4	0	1	
-5	4	0	
-6	0	0	
-7	1	0	
-8	1	0	
-9	0	0	
-10	0	0	
-NA	207	10	
**Previous SMM/MVA**			0.000206
-Yes	22	6	
-No	150	58	
-NA	104	12	
**Diagnosis Type**			0.0001898
-Missed	267	65	
-Incomplete	9	11	
**Missed Miscarriage CRL Median (range)(mm)**	6.0 (1.1–80.9)	7.0 (1.6–46.6)	0.5095
**Missed Miscarriage GS Median (range) (mm)**	21.9 (3.2–62.7)	25.3 (1.3–49.3)	0.01798
**Gestation of Miscarriage by USS Measurements**			0.1975
-5	5	0	
-6	67	15	
-7	48	12	
-8	28	9	
-9	15	8	
-10	3	2	
-11	3	1	
-12	2	0	
-NA	96	29	
**Gestation of Miscarriage by LMP**			0.9131
< 6	1	1	
-6	3	1	
-7	12	5	
-8	47	12	
-9	64	7	
-10	35	12	
-11	30	15	
-12	25	6	
-13	8	1	
->13	21	4	
-NA	30	13	

### Expectant management of miscarriage

In the expectant management study, training-validation and external test sets included 565 and 158 patients respectively. Patient demographics and clinical parameters are summarised in Table 1.

**Fig. 1:**
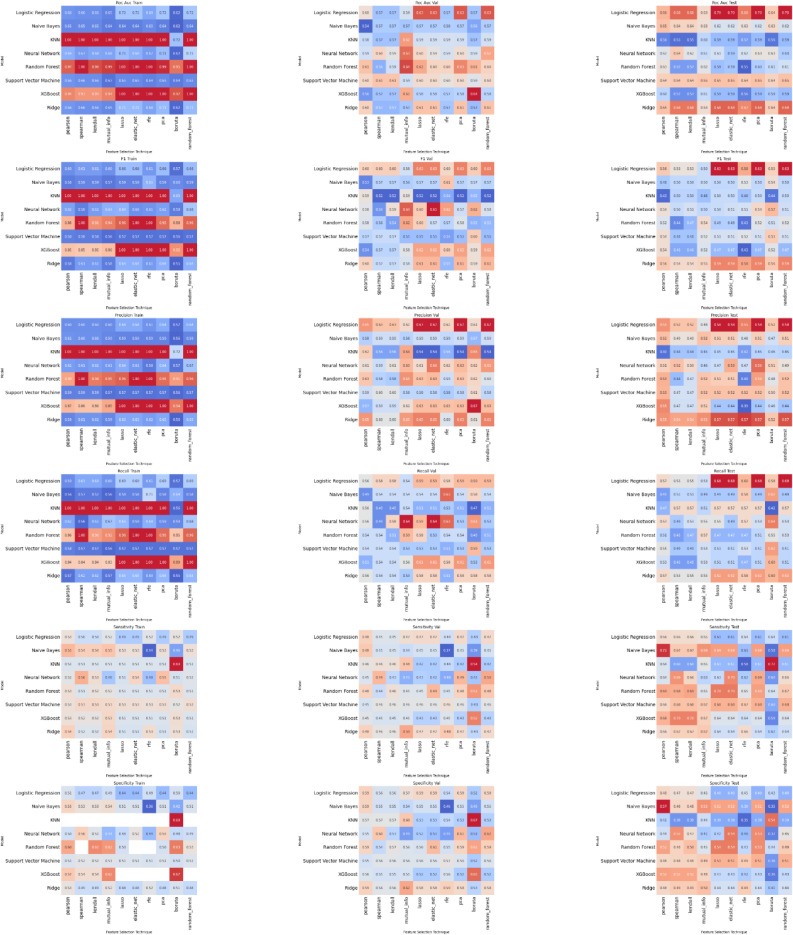
Predicting Outcome of Expectant Miscarriage Management. This figure includes a series of heatmaps, displaying the efficacy of various machine learning algorithms (displayed in columns) when combined with diverse feature reduction techniques (displayed in rows). Key for abbreviations: LASSO refers to Least Absolute Shrinkage and Selection Operator, E Net stands for Elastic-Net, RFE is Recursive Feature Elimination, Univariate LR corresponds to Univariate Logistic Regression, XGB represents Extreme Gradient Boosting Machine, NB is Naïve-Bayes, PSL corresponds to Partial Least Squares, L-SVM is Linear Support Vector Machine, NL-SVM is Non-linear (radial) SVM, RF refers to Random Forest, MDA denotes Mixture Discriminant Analysis, KNN signifies K-Nearest Neighbours, GLM is Generalised Linear Model, and NNET refers to Neural Network

Following data pre-processing a total of two continuous and 12 discrete features were used for machine learning. The results of our experiments with machine learning algorithms and feature set combinations on the validation set for each outcome are shown in Fig. 1. These are in the form of heatmaps showing the F1-score for each combination of machine learning algorithm (rows) with feature selection method (columns).

**Fig. 2:**
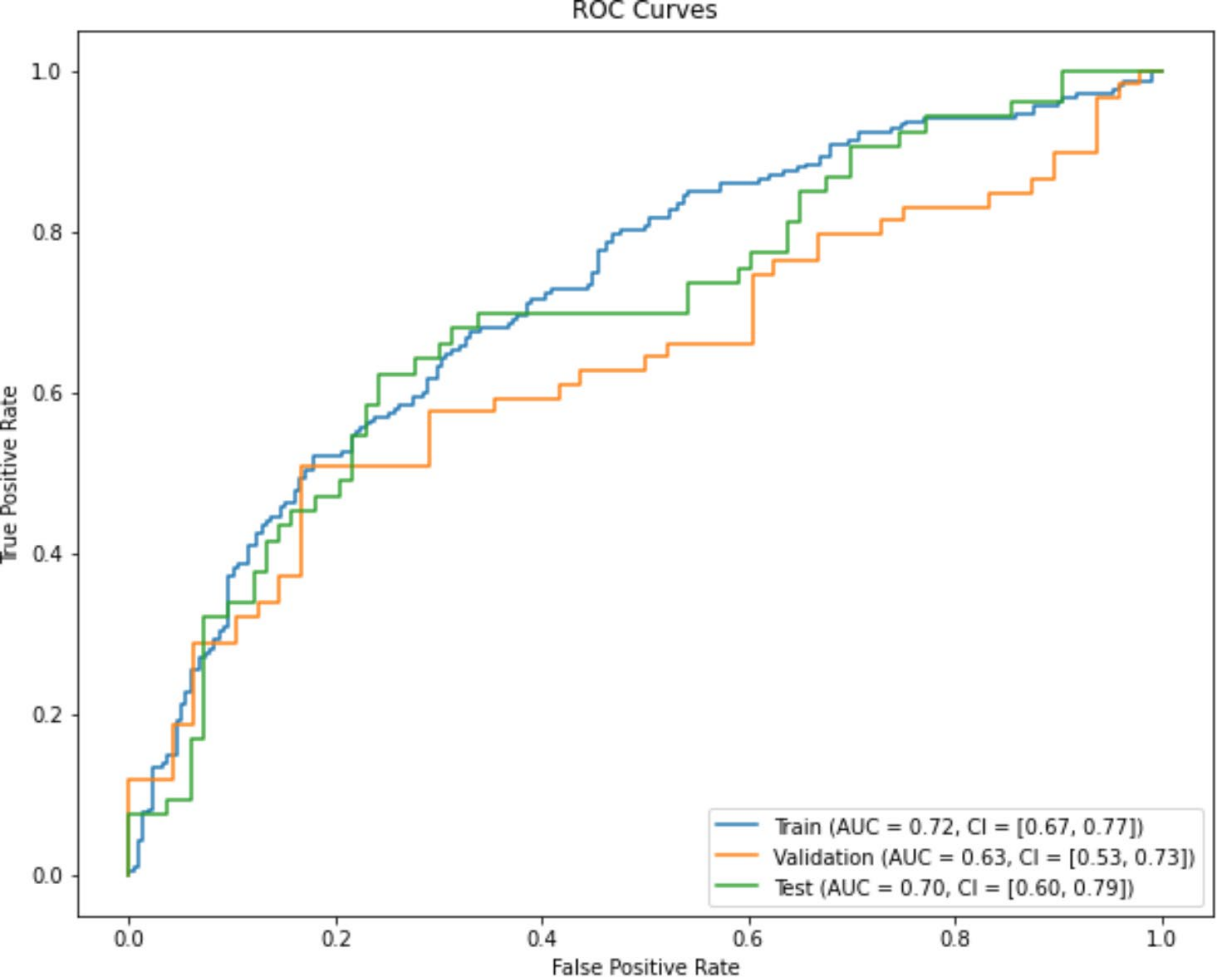
ROC curves for the validation and test set for expectant management. Training Set AUC = 0.72 (95% CI 0.67,0.77). Validation Set AUC = 0.63 (95% CI 0.53,0.73). External Test Set AUC = 0.70 (95% CI 0.60,0.79)

The final prediction model chosen for expectant management was Logistic Regression in combination with Least Absolute Shrinkage and Selection Operator (LASSO) feature reduction. Results of the final models on training, validation and external test sets are shown in Fig. 2 (ROC curves). The training set AUC = 0.72 (95% CI 0.67,0.77), validation set AUC = 0.63 (95% CI 0.53,0.73) and external test set AUC = 0.70 (95% CI 0.60,0.79). Performance of our models were consistent across training, validation and external test sets.

**Fig. 3:**
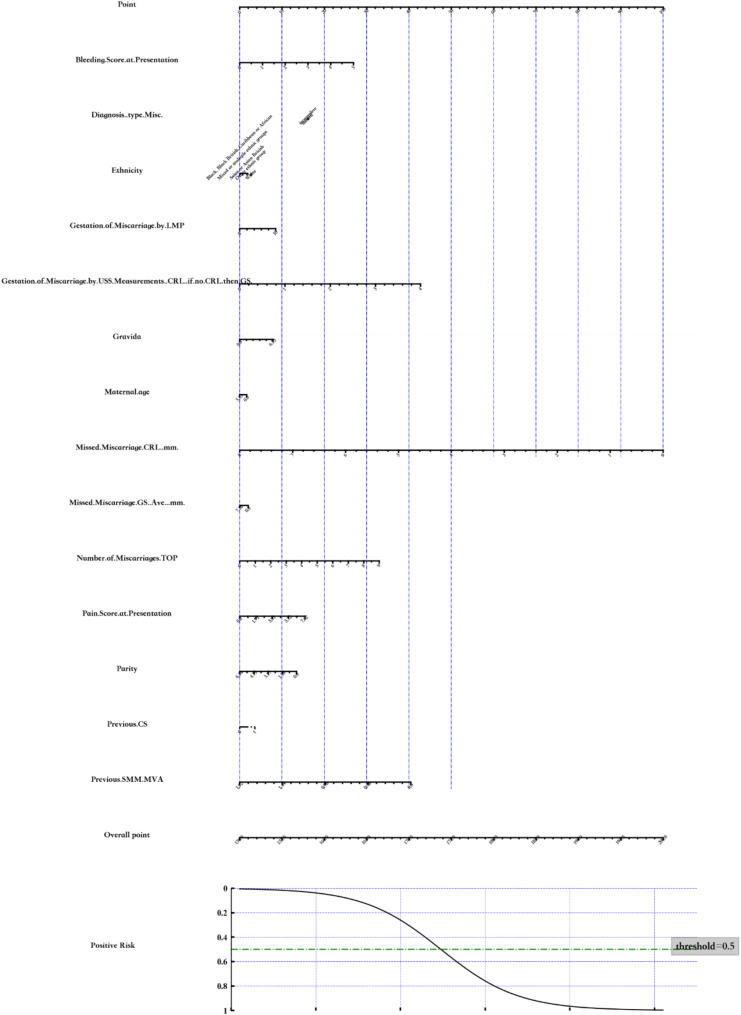
Nomogram: Expectant Management of Miscarriage

The expectant management predictive model has been converted into a nomogram for graphical representation, using the variable to estimate risk of success (Fig. 3).

### Medical management of miscarriage

In the medical management study, training-validation and external test sets included 276 and 76 patients respectively. Patient demographics and clinical parameters are summarised in Table 2.

**Fig. 4:**
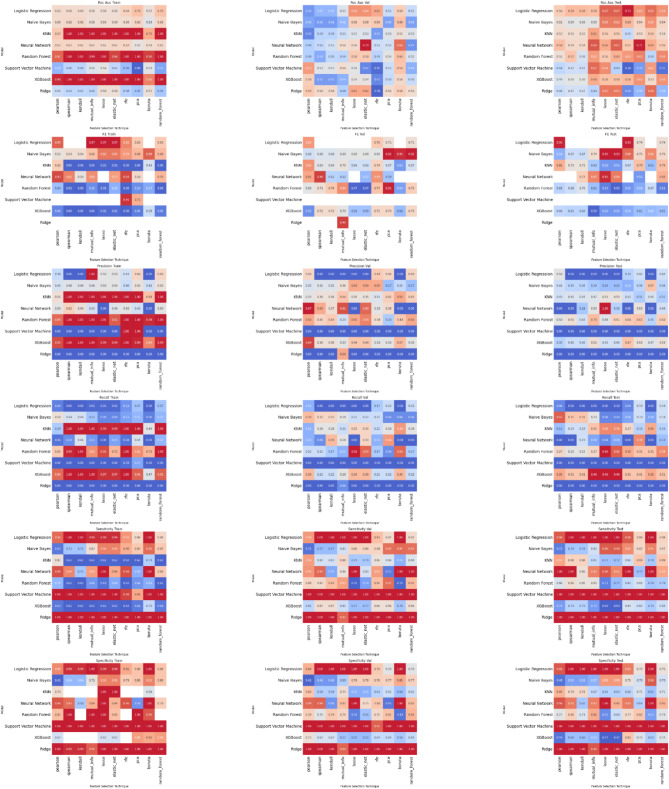
Predicting Outcome of Medical Miscarriage Management. Heatmaps illustrating the performance of each machine learning algorithm (columns) with each feature reduction method (rows). Key for abbreviations: LASSO refers to Least Absolute Shrinkage and Selection Operator, E Net stands for Elastic-Net, RFE is Recursive Feature Elimination, Univariate LR corresponds to Univariate Logistic Regression, XGB represents Extreme Gradient Boosting Machine, NB is Naïve-Bayes, PSL corresponds to Partial Least Squares, L-SVM is Linear Support Vector Machine, NL-SVM is Non-linear (radial) SVM, RF refers to Random Forest, MDA denotes Mixture Discriminant Analysis, KNN signifies K-Nearest Neighbours, GLM is Generalised Linear Model, and NNET refers to Neural Network

The same method of analysis was applied to the medical management cohort. Following data pre-processing a total of two continuous and 12 discrete features were used as input to the machine learning models. These are listed in Table 2, together with the features that remained after each feature reduction method for each of the measured outcomes. The results of our experiments using various combinations of machine learning algorithms and feature sets on the validation set for each outcome are shown in Fig. 4. These are in the form of heatmaps showing the F1-score for each combination of machine learning algorithm (rows) with feature selection method (columns).

**Fig. 5:**
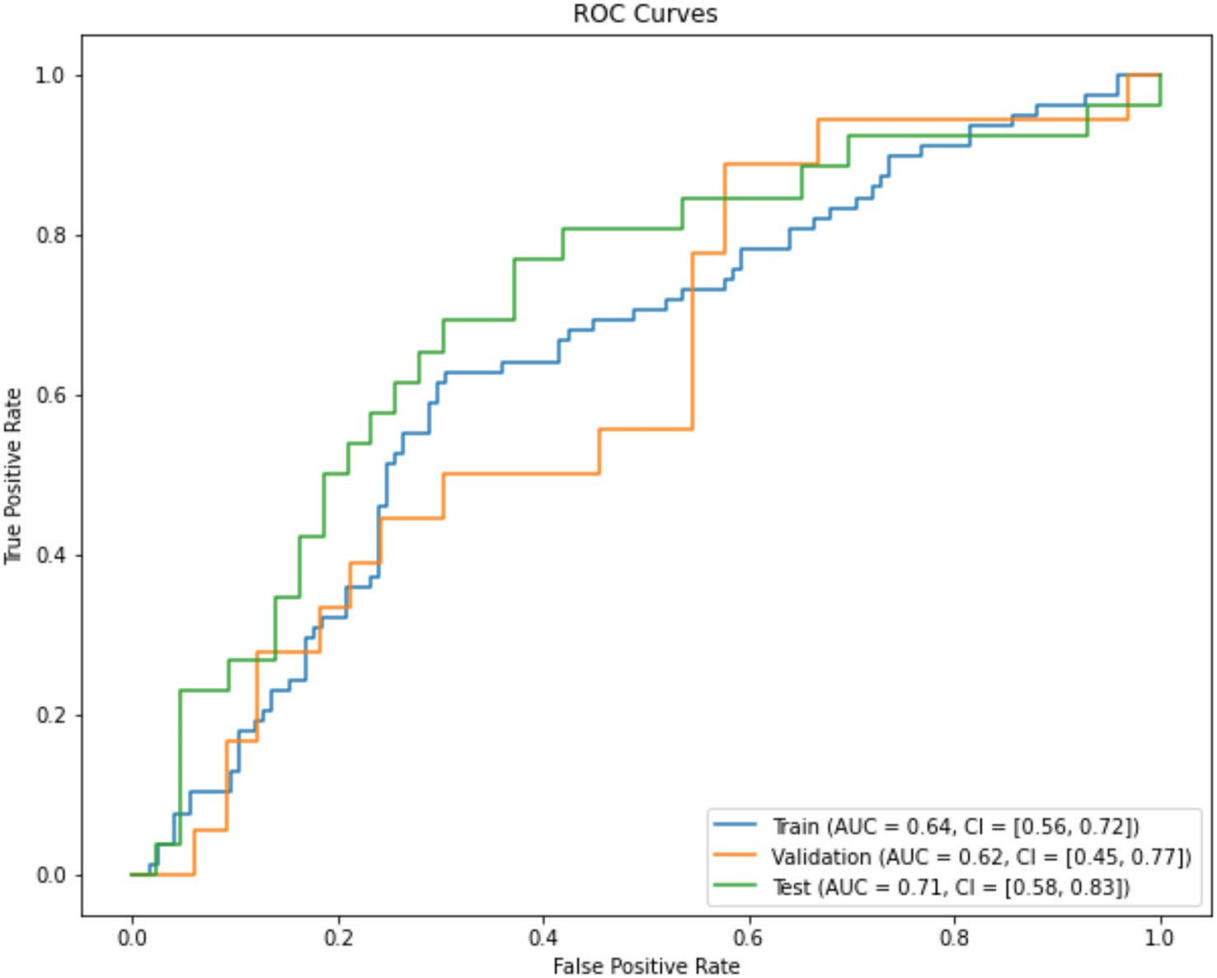
ROC curves for the training, validation and test set for medical management. Training Set AUC = 0.64 (95% CI 0.56,0.72). Validation Set AUC = 0.62 (95% CI 0.45,0.77). External Test Set AUC = 0.71 (95% CI 0.58,0.83)

The final prediction model chosen for medical management was Logistic Regression in combination with Recursive Feature Elimination (RFE) feature reduction. Results of the final models on training, validation and external test sets are shown in Fig. 5 (ROC curves). The training set AUC = 0.64 (95% CI 0.56,0.72), validation set AUC = 0.62 (95% CI 0.45,0.77) and external test set AUC = 0.71 (95% CI 0.58,0.83). Performance of our models were consistent across training, validation and external test sets.

**Fig. 6:**
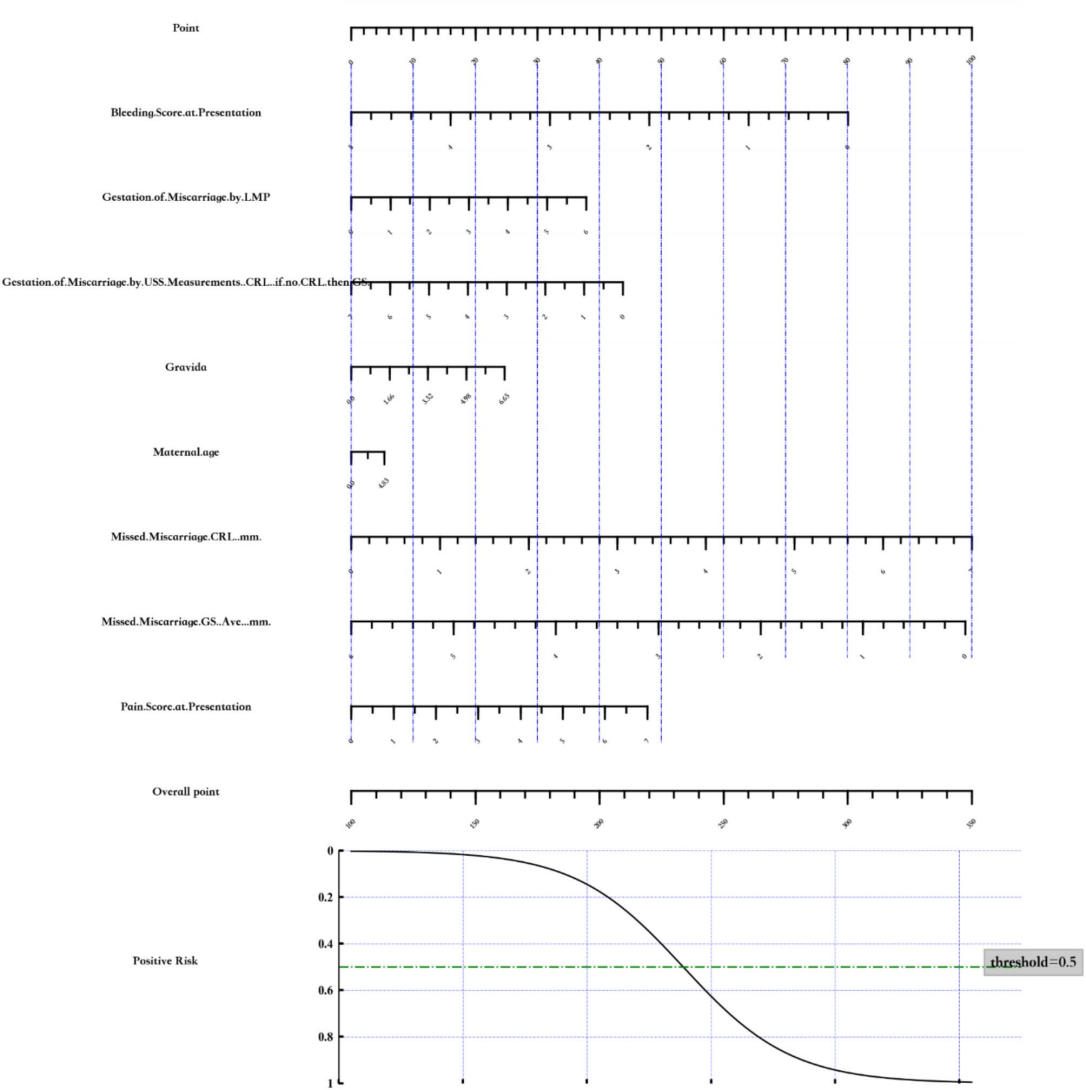
Nomogram: Medical Management of Miscarriage

The medical management predictive model has been converted into a nomogram for graphical representation, using the variable to estimate risk of success (Fig. 6).

### Feature importance

**Fig. 7:**
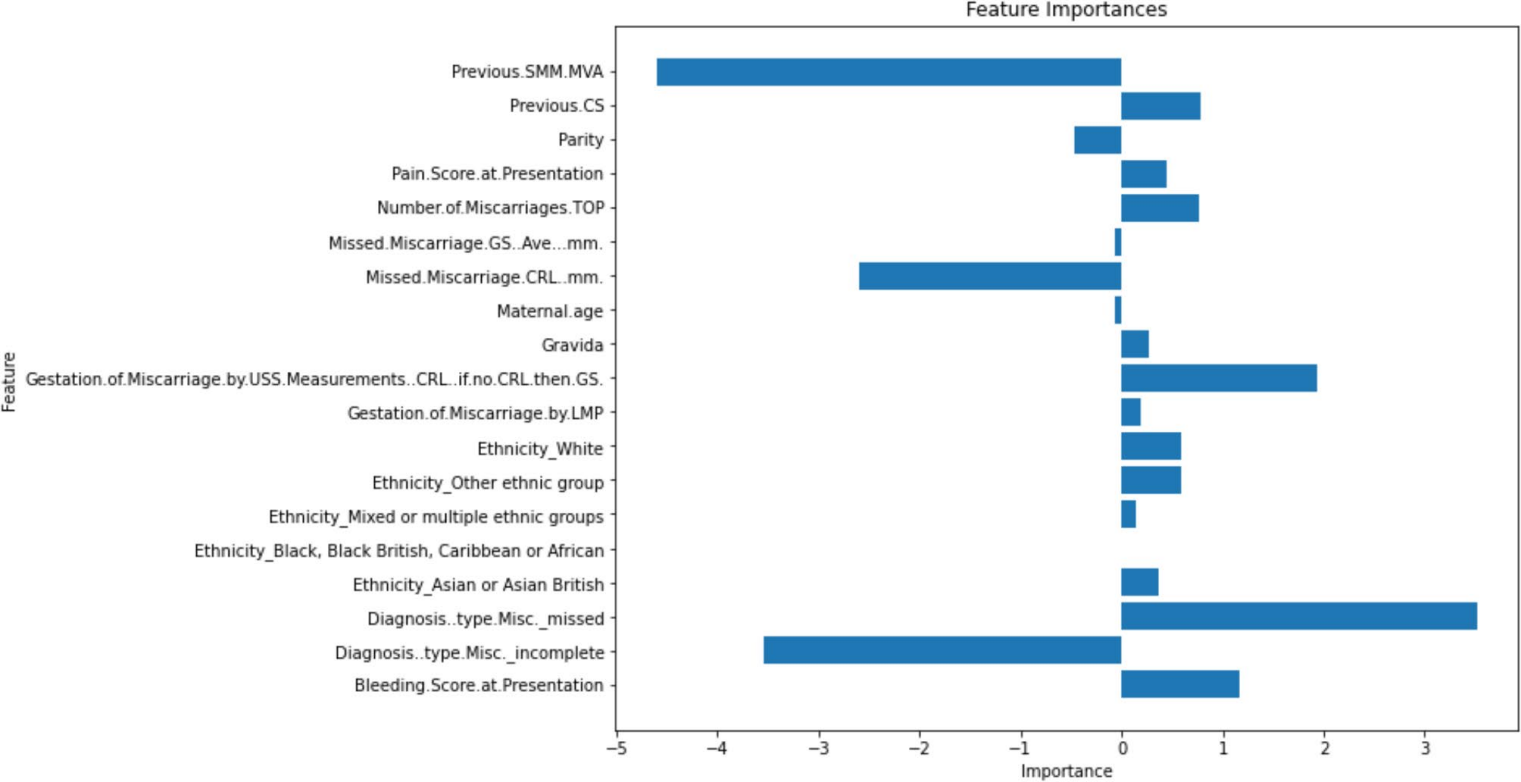
Least Absolute Shrinkage and Selection Operator (LASSO) feature reduction feature reduction method used in combination with the logistic regression [21] model: Expectant Management of Miscarriage

**Fig. 8:**
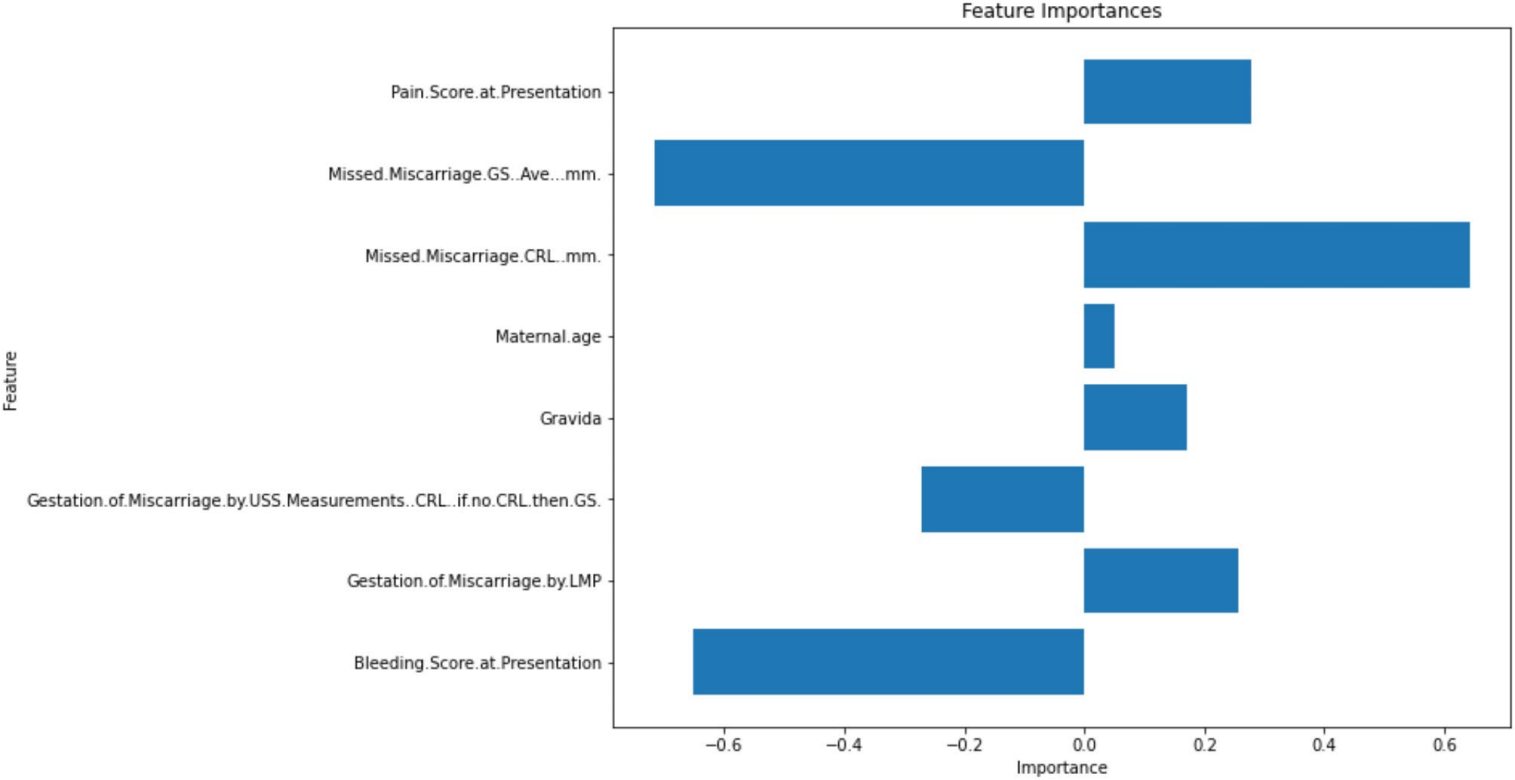
Recursive Feature Elimination (RFE) feature reduction method used in combination with the logistic regression [21] model: Medical Management of Miscarriage

Figure 7 outlines the feature importance results from the LASSO feature reduction method used in combination with the LR model, for expectant management of miscarriage. Figure 8 outlines the feature importance results from the RFE feature reduction method used in combination with the LR model, for medical management of miscarriage.

## Discussion

We have developed two machine learning models that can predict the outcomes from expectant and medical management of miscarriage respectively. This is the first multi-site UK study, with over 1000 patients, to systematically apply multiple ML techniques and feature reduction techniques in the development of a high performing, robust ML-based model to predict the outcome of expectant and medical management of miscarriage. The models had a consistent performance between the validation and external test sets, demonstrating generalisability which is important from a clinical utility perspective. These models are undoubtedly better than the current practice of using generalised outcome prediction for all women. These models could offer a personalised approach to care, through providing individualised outcome prediction and thus support the decision making process for the management of miscarriage.

For predicting expectant management outcomes the best performing model was the Logistic Regression model, with the Least Absolute Shrinkage and Selection Operator (LASSO) feature reduction method. There were five features which weighed most significantly with a feature importance score of > 1 or <-1 (Fig. 7). These features were: bleeding score at presentation, miscarriage diagnosis type (missed or incomplete), CRL (mm), gestation by ultrasound scan (USS) measurement and previous surgical management of miscarriage (SMM)/manual vacuum aspiration (MVA). The majority of these features demonstrate that the key predictors to successful expectant management within two weeks are indications that the process of miscarriage has already begun and size of the pregnancy. For example, a woman with pain, bleeding and incomplete miscarriage findings on USS is more likely to have successful expectant management, compared to a woman who is completely asymptomatic and the diagnosis from an incidental ultrasound is missed miscarriage. Ultimately, the best performing expectant management ML model required input of all the features collected. This feature set is relatively small (*n* = 14) using routinely collected clinical data, thus adds particular value as a user friendly tool to input data for a personalised outcome prediction.

In contrast, the best performing medical management outcome prediction model was the Logistic Regression model, with the Recursive Feature Elimination (RFE) feature reduction, using eight features. Features included a combination of ultrasound size of pregnancy, gestation duration based on both last menstrual period and ultrasound measurements, symptoms (bleeding and pain), maternal age and past obstetric history. This model, being a tree-based method, has its own internal regularisation with a number of estimators, and thus an inherent ability to handle multidimensional data and collinearity.

In the medical management predictive model, the important variables related to pregnancy size and length, might be expected given this cohort of patients, where the protocol of medical management is consistent in the first trimester, despite the variation in gestation and size of pregnancy tissue. The model used eight clinical features, which again is a relatively low number of features which should facilitate the use of this decision support tool to generate personalised statistics to guide miscarriage management.

In the literature, only two previous studies have developed models incorporating multiple features in order to predict miscarriage management success [15, 16]. Casikar 2013 developed a logistic regression model for the prediction of successful expectant management of first trimester miscarriage [15]. Multivariate analysis of clinical and ultrasonographic features, led to the three final independent prognostic variable used withing their model: type of miscarriage, vaginal bleeding and maternal age. Across these three clinical variables, the type of miscarriage and vaginal bleeding at presentation were the most significant in predicting success across the training and test sets. Casikar 2013, findings correlate with the features identified as important in our study following the application and comparison of multiple machine learning algorithms and feature selection methods.

Hamel 2022, developed a clinical prediction model for the success of medical management or early pregnancy loss [16]. Similarly to Casikar et al., 2013, multivariable logistic regression model was the only method of analysis. Selection of variables were determined by those with a p-value of below 0.157 as per the Akaike Information Criterion [24]. Interestingly, this study developed a model for predicting the success of medical management, included three clinical features not included in our study. Not included in our study were BMI, number of previous uterine aspirations and mifepristone pre-treatment, as these are not routinely collected data, so would restrict the practical application of the model. Furthermore, the number of uterine aspirations relies on patient recollection and understanding of this definition may not be consistent. The chosen regime for medical management is pre-determined by the provider and thus we did not include this as a variable. The aim of this study was to develop two ML models that have clinical value as a decision support tool. The systematic approach to model development has ensured the selection of the best performing predictive model, which is applicable and generalisable as demonstrated by the consistent results in the external datasets. The application and comparison of multiple machine learning models and feature reduction methods, is a relative strength of our analysis and reduces the risk of over-fitting.

Other strengths include the multi-site design with a large cohort of over 1000 patients from two London teaching hospitals adhering to national guidance in clinical practice, and are therefore likely to be representative of other units in the UK. The consistent performance across validation and external datasets support its generalisability and clinical utility. The use of readily available clinical data with no requirement for access to, and complex pre-processing of imaging data will support its wide clinical application.

The weaknesses of our study include its retrospective nature, and reliance on data retrieved from the online documentation (Cerner and Astraia) of participating centres, which resulted in missing data points in some cases. To address this limitation, we employed multiple imputation using the MICE method, incorporating convergence checks and sensitivity analyses to ensure robustness. While missing data remains a limitation, it reflects the realities of clinical practice and enhances the model’s applicability to data collected in routine clinical workflows. The decision was made not to include any biochemical markers, which have been previously demonstrated to be correlated to outcomes [9, 25, 26]. In clinical practice, bloods are not routinely taken and if they are there is no uniform time point. This decision was to ensure the model was generalisable with wide clinical utility using features that are routinely collected in early pregnancy units.

Another limitation to acknowledge is the imbalance between outcomes. While we used an 80:20 stratified split to maintain outcome balance during random splitting of data into training and validation sets—ICL E1 (expectant management) and ICL M1 (medical management)—and subsequently applied the models to an independent test dataset (ICL E2 and ICL M2), this approach primarily reduces the risk of overfitting. However, it does not fully address biases related to the balance of binary outcomes (success or failure of expectant or medical management of miscarriage respectively). To mitigate this, we calculated the F1-score, a metric that accounts for both false positives and false negatives, to provide a more meaningful evaluation of model performance for the minority class.

The benefits that these models offer are in being used as a clinician decision support tool, which can aid counselling to guide patients away from expectant or medical management if it is unlikely to be successful. Miscarriage has a significant impact on the emotional and psychological health on individuals and families affected. It has been demonstrated that miscarriage can trigger a mourning process similar to that experienced after death of a loved one [27]. For some individuals, the grief may persist for months or even years, affecting their emotional well-being and quality of life [28, 29]. The process of additionally dealing with the decision surrounding management choices, is likely an additional stressor, which may have a longer term impact on the psychological sequalae of miscarriage. Optimising the counselling of women through personalised guidance will have a positive impact.

Another consideration of the value in using these prediction models is the economic impact of unsuccessful management pathways. The short-term national economic cost of miscarriage has been estimated to be approximately £471 million per year in the UK [1]. A better understanding of individualised miscarriage management outcome prediction will lead to a reduction in the number of women who experience a prolonged management pathway due to failed management ultimately leading to surgery. This will have an impact on the economic burden of miscarriage by reducing time off work for appointments, lost productivity [30] and the impact on workforce participation [31]. Further to this the grief and emotional distress following a miscarriage may require counselling, therapy or other forms of mental health support, which is likely exacerbated in situations where the management is protracted. These services incur costs and may lead to reduced productivity in the workplace [31].

In this study we have compared multiple machine learning algorithms and feature selection methods to develop prediction models for percentage chance of success of expectant and medical management of first trimester miscarriage. Our models are novel in that their arrival has involved the collection of a large data set of features, chosen following thorough literature review and the trial of multiple machine learning algorithms to arrive at the highest performing model. This by far outweighs current clinical practice where there is a lack of personalised statistics to support decision making in miscarriage management. The models show consistency across validation and external test sets. There is value in the development of these models with the use of routinely available clinical data. This robust machine learning method, validated and externally tested, sets the stage for future clinical trials evaluating quantitative personalised statistics to guide clinicians and patients in deciding their miscarriage management plan.

## Electronic supplementary material

Below is the link to the electronic supplementary material.

Supplementary Material 1

## Data Availability

Due to confidentiality, data collected for the study are not publicly available for download due to ethical restrictions, however the corresponding authors can be contacted for academic enquiries on reasonable request.
