# Supplementary material for: Predicting outcomes of expectant and medical management in early pregnancy miscarriage using machine learning to develop and validate multivariable clinical prediction models
Source: BMC Pregnancy Childbirth. 2025 Feb 28;25:225. doi: 10.1186/s12884-025-07283-y (PMC11869538; doi:10.1186/s12884-025-07283-y)
Supplement: Supplementary file 1 — Supplementary Material 1 [file 12884_2025_7283_MOESM1_ESM.docx]

**Supplementary Material:**

**Table of Contents:**

Summary of machine learning algorithms

Summary of feature selection methods

Supplementary Table 1: Model Hyperparameters

Supplementary Table 2: Expectant Management: Results of classification for the validation and external test sets.

Supplementary Table 3: Medical Management: Results of classification for the validation and external test sets.

Supplementary Table 4: Comparison of previously published Miscarriage Management Outcome Prediction Machine Learning Models

Tripod Checklist

**Summary of machine learning algorithms:**

The models used in this study are supervised classification algorithms. A summary of each is provided below.

Logistic Regression - GLM (generalised linear model) is a generalisation of linear regression to modelling dependencies between predictors and dependent features. Logistic regression is a form of GLM used in this study. It uses the logistic sigmoid function to return a probability value which can then be mapped to two or more separate classes [^31^](#_ENREF_31).

Naïve Bayes (NB) – the Naïve-Bayes model is based on Bayes-theorem and assumes no interdependence between variables [^32^](#_ENREF_32).

KNN - K-nearest neighbours assumes that data points that are close to each other are of the same class. It takes a defined number (k) of training samples closest in Euclidian distance to a new point and predicts a class based on these [^33^](#_ENREF_33).

Neural Network – the “nnet” package fits a single-layer feed-forward neural network [^34^](#_ENREF_34).

RF & XGB – Random Forest and Extreme Gradient Boosting Machines are ensemble decision-tree based models. RF uses bagging and feature variability when building each decision tree to create an uncorrelated forest whose overall prediction is more accurate than each individual tree [^35^](#_ENREF_35). XGB by contrast takes a boosting approach whereby trees are grown iteratively using information from a previously grown tree, to minimise the error of previous trees [^36^](#_ENREF_36).

Support Vector Machine - SVM (linear and radial) – support vector machines plot training samples and assigns a hyperplane (decision boundary) to separate these into classes. The optimal hyperplane is that which maximises the distance between data-points. Where data is non-linear it is transformed to a higher dimensional feature space and a non-linear (e.g. radial) decision boundary is then applied to separate classes [^37^](#_ENREF_37).

Ridge regression is a regularisation method. Here a penalty is applied to the coefficient which multiplies each feature in a linear model and results in less overfitting and improved generalisation. Ridge uses the L2 regularisation penalty to force some coefficients to zero. Alpha = 0.5. The glmnet package was used to perform Ridge regression [^38^](#_ENREF_38).

**Summary of feature reduction methods:**

Pearson, Spearman’s rank and Kendall’s rank correlation are “filter” feature selection methods which rely only on the characteristics of feature independently of any machine learning model. Pearson’s correlation assumes data is parametric and linear. Spearman and Kendall’s rank are non-parametric and assume a monotonic relationship between variables. Kendall’s rank is preferred to Spearman’s where dataset have a limited number of observations or contain outliers. For our study we used the corr package and specified that the top 25%features be included in the feature sets following Pearson’s, Spearman’s and Kendall’s rank correlation (Venables WN, 2002).

MIM – Mutual Information Maximization feature reduction method measures the statistical dependence between two random variables. Mutual information quantifies the amount of information that one variable provides about another variable. In the context of feature selection, mutual information is used to evaluate the relationship between each feature and the target variable. MIM ranks features based on their mutual information with the target variable and selects the top-ranked features as the most relevant. By maximizing the mutual information, the MIM method aims to retain the features that have the highest predictive power or discriminative ability for the target variable [^39^](#_ENREF_39).

E-Net (Elastic Net Regression) and LASSO (Least Absolute Shrinkage and Selection Operator) are examples of regularisation methods [^38^](#_ENREF_38).. Here a penalty is applied to the coefficient which multiplies each feature in a linear model and results in less overfitting and improved generalisation. LASSO uses the L1 regularisation penalty to force some coefficients to zero. This eliminates some features leaving a subset of predictors that are thought to be important. Alpha = 1. Ridge uses the L2 regularisation penalty to force some coefficients to zero. Alpha = 0.5.Elastic Net incorporates penalties from both L1 and L2 (ridge regression) regularisation. The glmnet package was used to perform LASSO, Ridge and Elastic-net regression.

RFE – Recursive Feature Elimination is a “wrapper” feature selection method which fits a model and removes the weakest feature until a specified number of features is reached. Cross-validation is used to score different feature subsets and select the best scoring collection of features to identify the optimal number of features. Features are ranked by the model’s feature importance. By recursively eliminating features iteratively the collinearity is reduced (Guyon 2002). We used the rfeControl package with a random forest model and 10-fold cross validation with 5 repeats.

Principal Component Analysis (PCA): A dimensionality reduction technique that transforms the original features into a new set of orthogonal features called principal components. These components capture the maximum variance in the data.

Boruta – Designed as a wrapper around a Random Forest classifier, which systematically removes features demonstrated to be less relevant than random probes by means of statistical testing.

Random Forest as described above, calculates feature importance scores based on how much each feature contributes to the overall performance of the ensemble.

**Supplementary Table 1: Model Hyperparameters**

| **Model** | **Hyperparameters** |
| --- | --- |
| **Logistic Regression** | Penalty: {L1, L2}; Regularization strength (C): {0.01, 0.1, 1, 10, 100}; Solver: {liblinear, saga} |
| **Naive Bayes** | Not applicable |
| **K-Nearest Neighbors** | Number of neighbors (k): {3, 5, 10, 20, 30}; Weights: {Uniform, Distance}; Metric: {minkowski, euclidean, manhattan} |
| **Neural Network** | Hidden layer sizes: {(10,), (20,), (50,), (100,)}; Activation: {ReLU, tanh, logistic}; Alpha (Regularization term): {0.0001, 0.001, 0.01, 0.1}; Solver: {adam, lbfgs, sgd} |
| **Random Forest** | Number of trees (n): {100, 200, 500}; Maximum depth: {5, 10, 20, None}; Minimum samples per split: {2, 5, 10}; Max features: {sqrt, log2, None} |
| **Support Vector Machine** | Regularization strength (C): {0.01, 0.1, 1, 10, 100}; Kernel coefficient (gamma): {0.01, 0.1, 1, scale, auto}; Kernel: {linear, rbf, poly, sigmoid} |
| **XGBoost** | Number of estimators: {100, 200, 500}; Learning rate: {0.01, 0.05, 0.1, 0.2}; Maximum depth: {3, 5, 7, 10}; Subsample: {0.6, 0.8, 1.0}; Colsample_bytree: {0.6, 0.8, 1.0} |
| **Ridge Regression** | Regularization strength (Alpha): {0.01, 0.1, 1, 10, 100} |

**Supplementary Table 2:** **Expectant Management:** Results of classification for the validation and external test sets.

| **Metric** | **Validation** | **Test** |
| --- | --- | --- |
| **Accuracy** | 0.60 | 0.67 |
| **Balanced Accuracy** | 0.60 | 0.66 |
| **F1 Score** | 0.610 | 0.60 |
| **Sensitivity / Recall** | 0.58 | 0.62 |
| **Specificity** | 0.63 | 0.70 |
| **PPV / Precision** | 0.65 | 0.57 |
| **Brier Score** | 0.25 | 0.22 |
| **Accuracy 95% CI** | [0.50, 0.69] | [0.59, 0.75] |

**Supplementary Table 2:** **Medical Management:** Results of classification for the validation and external test sets.

| **Metric** | **Validation** | **Test** |
| --- | --- | --- |
| **Accuracy** | 0.63 | 0.61 |
| **Balanced Accuracy** | 0.53 | 0.50 |
| **F1 Score** | 0.73 | 0.94 |
| **Sensitivity / Recall** | 0.83 | 0.04 |
| **Specificity** | 0.88 | 0.95 |
| **PPV / Precision** | 0.43 | 0.33 |
| **Brier Score** | 0.23 | 0.22 |
| **Accuracy 95% CI** | [0.49, 0.75] | [0.49, 0.72] |

**Supplementary Table 4: Comparison of previously published Miscarriage Management Outcome Prediction Machine Learning Models**

| **Author, Year** | **Management Type** | **Cohort Size** | **ML Method** | **Validation/External Test** | **AUC Score** |
| --- | --- | --- | --- | --- | --- |
| Murugesu, 2025 | Expectant | 723 | 8 different Machine Learning Models alongside 10 Feature Reduction Methods. Final Combination: MIM/KNN | Internal validation and External Test Set from alternative early pregnancy unit located at a different hospital site | 0.70 (95% CI 0.60,0.79) |
| Casikar, 2013 | Expectant | 312 | Multivariable logistic regression model | Prospective validation, cohort from same early pregnancy unit | 0.796 |
| Murugesu, 2023 | Medical | 352 | 8 different Machine Learning Models alongside 10 Feature Reduction Methods. Final Combination: Kendall/RF | Internal validation and External Test Set from alternative early pregnancy unit located at a different hospital site | 0.71 (95% CI 0.58,0.83) |
| Hamel, 2022 | Medical | 344 | Multivariable logistic regression model | Internal validation applied by bootstrapping | 0.68 (95% CI 0.65, 0.70) |

**Tripod Checklist:**

| **Section/Topic** | **Item** |  | **Checklist Item** | **Page** |
| --- | --- | --- | --- | --- |
| **Title and abstract** | | | | |
| Title | 1 | D;V | Identify the study as developing and/or validating a multivariable prediction model, the target population, and the outcome to be predicted. | X |
| Abstract | 2 | D;V | Provide a summary of objectives, study design, setting, participants, sample size, predictors, outcome, statistical analysis, results, and conclusions. | X |
| **Introduction** | | | | |
| Background and objectives | 3a | D;V | Explain the medical context (including whether diagnostic or prognostic) and rationale for developing or validating the multivariable prediction model, including references to existing models. | X |
|  | 3b | D;V | Specify the objectives, including whether the study describes the development or validation of the model or both. | X |
| **Methods** | | | | |
| Source of data | 4a | D;V | Describe the study design or source of data (e.g., randomized trial, cohort, or registry data), separately for the development and validation data sets, if applicable. | X |
|  | 4b | D;V | Specify the key study dates, including start of accrual; end of accrual; and, if applicable, end of follow-up. | X |
| Participants | 5a | D;V | Specify key elements of the study setting (e.g., primary care, secondary care, general population) including number and location of centres. | X |
|  | 5b | D;V | Describe eligibility criteria for participants. | X |
|  | 5c | D;V | Give details of treatments received, if relevant. | X |
| Outcome | 6a | D;V | Clearly define the outcome that is predicted by the prediction model, including how and when assessed. | X |
|  | 6b | D;V | Report any actions to blind assessment of the outcome to be predicted. |  |
| Predictors | 7a | D;V | Clearly define all predictors used in developing or validating the multivariable prediction model, including how and when they were measured. | X |
|  | 7b | D;V | Report any actions to blind assessment of predictors for the outcome and other predictors. |  |
| Sample size | 8 | D;V | Explain how the study size was arrived at. |  |
| Missing data | 9 | D;V | Describe how missing data were handled (e.g., complete-case analysis, single imputation, multiple imputation) with details of any imputation method. | X |
| Statistical analysis methods | 10a | D | Describe how predictors were handled in the analyses. | X |
|  | 10b | D | Specify type of model, all model-building procedures (including any predictor selection), and method for internal validation. | X |
|  | 10c | V | For validation, describe how the predictions were calculated. | X |
|  | 10d | D;V | Specify all measures used to assess model performance and, if relevant, to compare multiple models. | X |
|  | 10e | V | Describe any model updating (e.g., recalibration) arising from the validation, if done. |  |
| Risk groups | 11 | D;V | Provide details on how risk groups were created, if done. | X |
| Development vs. validation | 12 | V | For validation, identify any differences from the development data in setting, eligibility criteria, outcome, and predictors. | X |
| **Results** | | | | |
| Participants | 13a | D;V | Describe the flow of participants through the study, including the number of participants with and without the outcome and, if applicable, a summary of the follow-up time. A diagram may be helpful. | X |
|  | 13b | D;V | Describe the characteristics of the participants (basic demographics, clinical features, available predictors), including the number of participants with missing data for predictors and outcome. | X |
|  | 13c | V | For validation, show a comparison with the development data of the distribution of important variables (demographics, predictors and outcome). | X |
| Model development | 14a | D | Specify the number of participants and outcome events in each analysis. | X |
|  | 14b | D | If done, report the unadjusted association between each candidate predictor and outcome. |  |
| Model specification | 15a | D | Present the full prediction model to allow predictions for individuals (i.e., all regression coefficients, and model intercept or baseline survival at a given time point). |  |
|  | 15b | D | Explain how to the use the prediction model. |  |
| Model performance | 16 | D;V | Report performance measures (with CIs) for the prediction model. | X |
| Model-updating | 17 | V | If done, report the results from any model updating (i.e., model specification, model performance). |  |
| **Discussion** | | | | |
| Limitations | 18 | D;V | Discuss any limitations of the study (such as nonrepresentative sample, few events per predictor, missing data). | X |
| Interpretation | 19a | V | For validation, discuss the results with reference to performance in the development data, and any other validation data. | X |
|  | 19b | D;V | Give an overall interpretation of the results, considering objectives, limitations, results from similar studies, and other relevant evidence. | X |
| Implications | 20 | D;V | Discuss the potential clinical use of the model and implications for future research. | X |
| **Other information** | | | | |
| Supplementary information | 21 | D;V | Provide information about the availability of supplementary resources, such as study protocol, Web calculator, and data sets. | X |
| Funding | 22 | D;V | Give the source of funding and the role of the funders for the present study. | X |

*Items relevant only to the development of a prediction model are denoted by D, items relating solely to a validation of a prediction model are denoted by V, and items relating to both are denoted D;V. We recommend using the TRIPOD Checklist in conjunction with the TRIPOD Explanation and Elaboration document.

**References**

31. Venables WN RB. Generalized Linear Models. In: Modern Applied Statistics with S. . New York, NY: Springer New York; 2002.

32. Majka M. High performance implementation of the Naive Bayes algorithm. R package naivebayes. Version 0.9.7. 2019. 2021 (accessed 13 August 2021).

33. Venables WN RB. Classification. In: Modern Applied Statistics with S. New York, NY: Springer New York; 2002: 331-51.

34. Riplley B VB, Bates DM, Firth D, Hornik K, Gebhardt A. Package ‘MASS’. Support Functions and Datasets for Venables and Ripley’s MASS. 20182022).

35. Breiman L. Random Forests. *Machine Learning* 2001; **45**(1): 5-32.

36. Chen TQ GC. Xgboost: A Scalable Tree Boosting System. Proceedings of the 22nd ACM SIGKDD International Conference on Knowledge Discovery and Data Mining. San Francisco; 2016. p. 785-94.

37. Karatzoglou A, Smola A, Hornik K, Zeileis A. kernlab-an S4 package for kernel methods in R. *Journal of statistical software* 2004; **11**: 1-20.

38. Friedman J, Hastie T, Tibshirani R. Regularization Paths for Generalized Linear Models via Coordinate Descent. *J Stat Softw* 2010; **33**(1): 1-22.

39. Peng H, Fan Y. Feature selection by optimizing a lower bound of conditional mutual information. *Inf Sci (N Y)* 2017; **418-419**: 652-67.
